# *In Situ* Observation of Topotactic Linker Reorganization in the Aperiodic Metal–Organic Framework TRUMOF-1

**DOI:** 10.1021/jacs.4c09487

**Published:** 2024-09-26

**Authors:** Guy Greenbaum, Patrick W. Doheny, Robert A. I. Paraoan, Yevheniia Kholina, Stefan Michalik, Simon J. Cassidy, Hamish H.-M. Yeung, Andrew L. Goodwin

**Affiliations:** † Department of Chemistry, University of Oxford, Inorganic Chemistry Laboratory, Oxford OX1 3QR, U.K.; ‡ School of Chemistry, University of Birmingham, B15 2TT Birmingham, U.K.; ¶ Department of Materials, ETH Zürich, 8093 Zürich, Switzerland; § Diamond Light Source Ltd., Harwell Science and Innovation Campus, Didcot OX11 0DE, U.K.

## Abstract

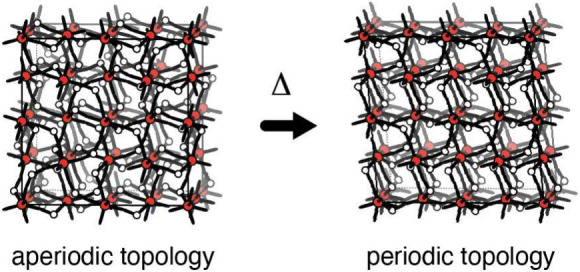

We use *in situ* synchrotron X-ray diffraction measurements to monitor the solvothermal crystallization mechanism of the aperiodic metal–organic framework TRUMOF-1. Following an initial incubation period, TRUMOF-1 forms as a metastable intermediate that subsequently transforms into an ordered product with triclinic crystal symmetry. We determine the structure of this ordered phase, which we call msw-TRUMOF-1, and show that it is related to TRUMOF-1 through topotactic reorganization of linker occupancies. Our results imply that the connectivity of TRUMOF-1 can be reorganized, as required for data storage and manipulation applications.

The unusual material TRUMOF-1 is a crystalline metal–organic framework (MOF) with an aperiodic network connectivity.^1^ Its structure is assembled from OZn_4_ nodes connected by 1,3-benzenedicarboxylate (1,3-bdc) linkers (inset to Figure 1a). The nodes are arranged on the vertices of a face-centered-cubic (**fcu**) lattice; each OZn_4_ unit is coordinated in an octahedral fashion by six 1,3-bdc linkers, and each linker connects two neighboring nodes. Because the underlying **fcu** net is 12-connected, only a subset of the many possible nearest-neighbor links can actually be occupied if octahedral coordination is preserved. So although TRUMOF-1 can be grown as single crystals with periodic node arrangements, its structure is nonetheless aperiodic because the linker occupancies are disordered.^2^ This disorder is not random,^3,4^ but follows a set of local rules that relates the structure of TRUMOF-1 to the broader family of Truchet tilings historically explored as visual information stores.^5,6^

**Figure 1:**
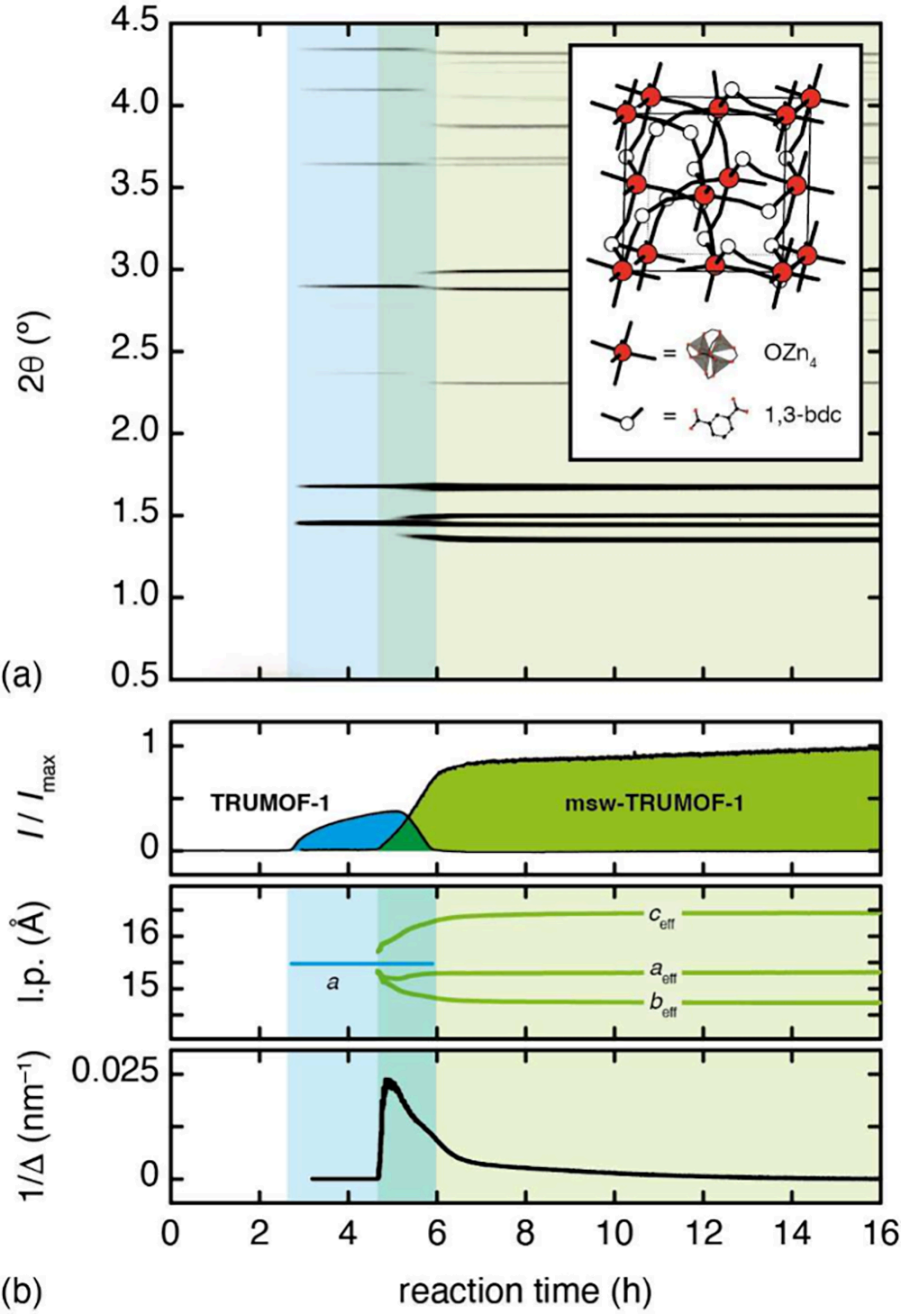
*In situ* synchrotron X-ray diffraction measurements of TRUMOF-1 formation and ordering in DMF at 110 °C. (a) The background-subtracted X-ray diffraction patterns (λ = 0.226 Å) show the emergence of TRUMOF-1 at about 3 h (blue shaded region) and its subsequent transformation into msw-TRUMOF-1 at about 5 h (green shaded region). The inset shows a simplified representation of one 1 × 1 × 1 approximant of the TRUMOF-1 structure: OZn_4_ clusters are coordinated octahedrally by six 1,3-bdc linkers, each of which bridges two clusters. (b) Variation in (top) relative phase intensities, (middle) effective lattice parameters (l.p.s), and (bottom) inverse crystallite size Δ extracted from sequential Pawley refinements.

Because TRUMOF-1 is unique among MOFs for its topological aperiodicity, we were interested in understanding how it actually forms during solvothermal synthesis. It is not obvious, for example, whether it evolves from an amorphous precursor, or indeed emerges from competition with other, more conventional, crystallization products. *In situ* X-ray diffraction, performed by probing a solvothermal reaction vessel with high-energy synchrotron X-ray radiation, is the method of choice for characterizing crystallization pathways^7−9^ and has provided key insight into the formation mechanisms of many canonical MOF families.^10−16^ Consequently we carried out a series of such measurements for the crystallization of TRUMOF-1 from its Zn/1,3-bdc precursors, following the synthetic strategy of ref (1); further details of our experiment are given as Supporting Information (SI).

Our key results are shown in Figure 1a for synthesis in *N*,*N*-dimethylformamide (DMF) at 110 °C. After an initial incubation period of ∼3 h involving only smooth variation in the small-angle and background scattering, Bragg reflections characteristic of the high-symmetry *F*4̅3*m* TRUMOF-1 structure appeared. The X-ray diffraction pattern in this regime could be modeled well using the single-crystal structure solution of ref (1), and the variation in integrated intensity with time accounted for using a modified Gaultieri model^13,17^ (see the SI). After ∼2 h, a more complex set of Bragg reflections emerged in the diffraction pattern, signifying a transition to a new structure type with lower (average) symmetry. This new phase grew at the expense of TRUMOF-1 and, after a brief coexistence period, was the only crystalline phase present thereafter. Similar behavior was observed in measurements carried out at 118 and 128 °C, except that the time scales involved were systematically reduced (see the SI). These variable-temperature data allowed us to estimate the activation energy barrier to nucleation for TRUMOF-1 as 61(3) kJ mol^–1^, which is typical for MOFs.^13,18^

The diffraction pattern of the final product shares a similar overall intensity profile with that of TRUMOF-1, suggesting that the underlying *F*4̅3*m* symmetry of the node arrangement should still be present in the new phase, but with symmetry broken by ordering of linker occupancies. The highest symmetry subgroup of *F*4̅3*m* in which we could index the final diffraction pattern corresponded to a monoclinic *Pm* cell with half the volume of the original cubic cell.^39^

A structural model using this *Pm* setting (with cell dimensions obtained from Pawley refinement) and the positional coordinates and occupancies of TRUMOF-1 gave an acceptable but not excellent Rietveld fit. The quality of this fit could be improved by refining linker occupancies and atom coordinates (as permitted in *Pm*), but the number of degrees of freedom (even when employing strict rigid-body and stoichiometric constraints) was simply too large to arrive at a unique, chemically sensitive structure solution from powder data.

A recurring feature among competing solutions, however, was the vanishing occupancy of 1,3-bdc linker orientations involving carboxylates pointing along one specific common direction. This direction lay within the mirror plane of the *Pm* cell, and inspection of the structure made clear that vacancies in this direction could rationalize the monoclinic shear observed experimentally. Consequently, we postulated that the symmetry-breaking transformation of TRUMOF-1 might be driven by cooperative reorganization of linkers to avoid one common carboxylate direction. To test this hypothesis, we used a Monte Carlo algorithm to identify what set of 1,3-bdc linker orientations might satisfy this constraint while also obeying the underlying TRUMOF-1 connectivity rules. We found that there is a unique simplest solution, described by an arrangement of linkers with the **msw** topology,^19^ giving triclinic *P*1 symmetry and a unit cell of dimensions similar to those of the *Pm* model discussed above; this solution is actually one of the 1 × 1 × 1 “approximants” to the TRUMOF-1 structure originally reported in ref (1) (Figure 2).

**Figure 2:**
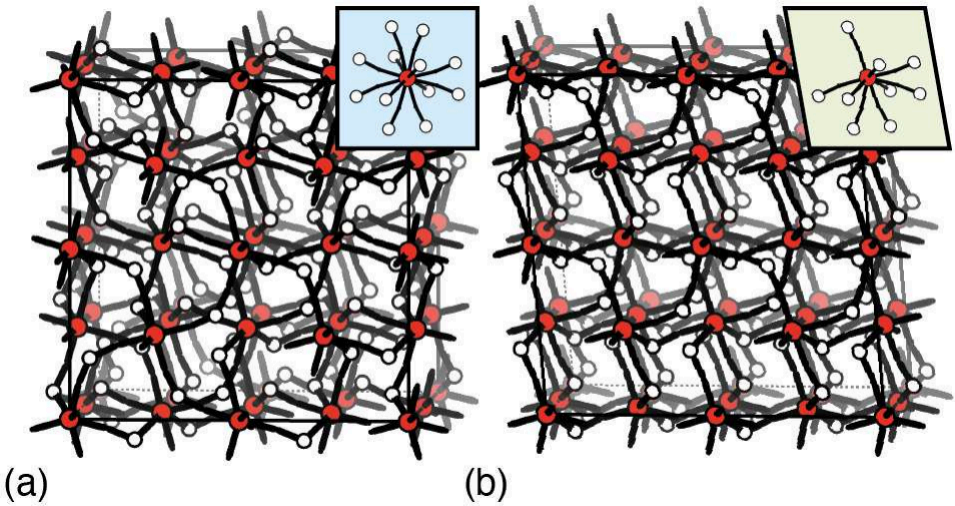
Topological order and disorder in (msw-)TRUMOF-1. (a) A 2 × 2 × 2 approximant of the TRUMOF-1 structure is shown using the representation of Figure 1a. Such approximants involve population of all 12 possible linker orientations (inset). (b) The topology of msw-TRUMOF-1 is the simplest that arises if any single linker orientation is forbidden. In fact the absence of one implies the absence of two others, such that only nine are populated in practice (inset). The uneven population of linker sites breaks the cubic crystal symmetry and allows structural relaxation through lattice shear.

Using the atomic coordinates obtained through DFT geometry optimization of the *P*1 approximant and the unit-cell dimensions obtained from our *Pm* Pawley refinements to construct a initial structural model, we carried out a Rietveld refinement against our newly acquired X-ray diffraction data. The fit obtained was good (*R*_wp_ = 2.9%) (Figure 3a), especially considering the complexity of the structural model involved and the highly constrained nature of our refinement. We used rigid-body constraints and bond-length/bond-angle restraints to ensure sensible chemical connectivity and to ensure that the number of free parameters was very low. The refined structural model, which is shown in Figure 3b, represents an orientationally ordered variant of TRUMOF-1 that we label msw-TRUMOF-1 (leaving open the possibility that other ordered variants with different topologies may also exist). While we cannot rule out the existence of other structure solutions to the powder X-ray diffraction data, we believe this model to be the simplest explainable in terms of (relatively) simple chemical rules. There is strong pseudosymmetry in this structure, which is why the triclinic unit-cell angles α and γ are so close to 90° (any deviation being immeasurable within the limits of our experimental resolution) and the diffraction pattern can be indexed in a monoclinic setting.

**Figure 3:**
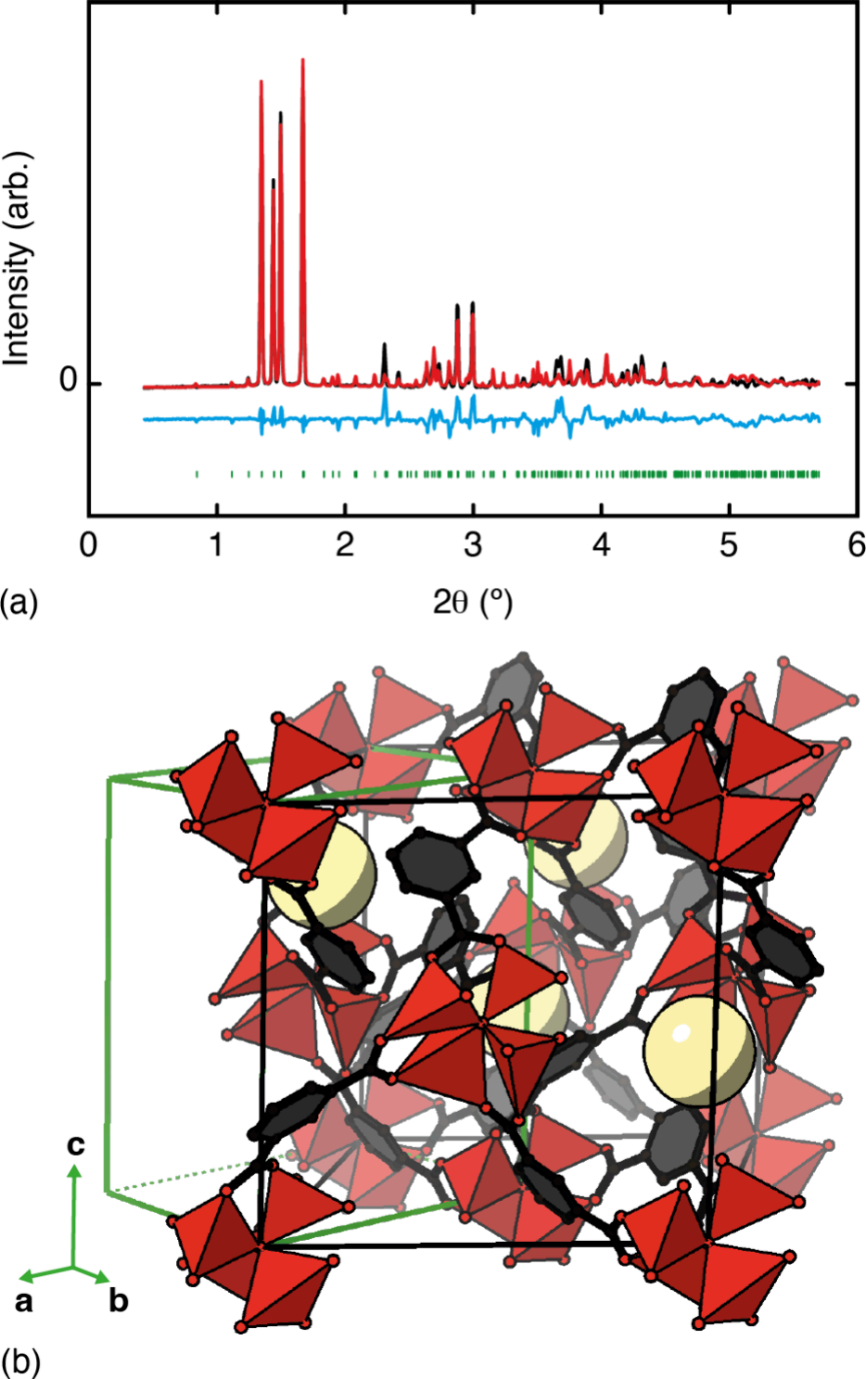
Structure determination of msw-TRUMOF-1. (a) The final background-subtracted X-ray diffraction pattern (black lines) and corresponding Rietveld fit (red lines) as described in the text. Reflection positions are shown as green tick marks, and the difference function (data – fit) is shown as a blue line, shifted vertically for clarity. (b) Representation of the Rietveld-refined structure of msw-TRUMOF-1 with Zn coordination polyhedra shown in red and C and O atoms as black and red spheres, respectively, and disordered solvent is shown as large yellow spheres. The *P*1 unit cell is shown in green; its volume is half that of the original *F*4̅3*m* cell (shown in black).

There are two primary mechanisms by which the transformation from TRUMOF-1 to msw-TRUMOF-1 might proceed: either dissolution/recrystallization or topotactic reorganization. The latter implies a registry between parent and daughter MOF lattices such that strain should initially be small in msw-TRUMOF-1 and increase as the transformation proceeds. Pawley refinements allowed us to track this process: we found that, on first formation, the msw-TRUMOF-1 lattice is indeed metrically close to that of TRUMOF-1 but that both symmetry-breaking strain and coherence length increase as transformation proceeds (Figure 1b). Were the process to involve homogeneous crystallization of msw-TRUMOF-1 from solution, we would expect the phase to grow with constant maximal strain throughout the experiment. Hence our data are consistent with a model in which linker-orientational order develops initially over small domains—the lack of coherence resulting in a relatively small splitting of the parent Bragg reflections. We cannot say whether these domains are buried in the interior of the TRUMOF-1 crystallites or emerge at their surfaces. One way or the other, these domains eventually coalesce and reorganize to drive a stronger cooperative strain that is now coherent over a larger length scale.

The central implication of our findings is that linker orientations in TRUMOF-1—and hence the particular Truchet tilings to which they correspond—are not fixed. Instead the system is able to explore its configurational landscape dynamically (at least at the elevated temperatures explored here) and in doing so arrive eventually at an enthalpically favored ordered state. The DFT calculations of ref (1) suggest this landscape is very shallow, with different TRUMOF-1 approximants differing by just a few kJ mol_Zn_^–1^—much less than the ∼80 kJ mol^–1^ energy scale of DMF–OZn_4_ interactions.^20^ Hence we anticipate reorganization involves the DMF-assisted breaking of carboxylate–OZn_4_ bonds,^21^ as implicated in the more general phenomenon of solvent-assisted ligand exchange.^22^

Truchet-tile structures are of interest in general terms for their ability to store information.^6^ In the context of TRUMOF-1, this information is contained within the connectivity of the framework structure itself. The initial assumption of ref (1) was that this connectivity was imprinted during synthesis, but we now find that extending the solvothermal regime modifies the information content of TRUMOF-1 crystallites. Such a process is necessary if systems such as TRUMOF-1 are ever to be exploited in data processing applications and opens up the possibility of using TRUMOF-1 reorganization in reservoir computing.^23^

The topotactic transformation we have observed is unusual in the broader context of aperiodic systems and may be the first of its kind. The topology of TRUMOF-1 is a form of continuous random network (CRN) structure,^24^ related to those of conventional amorphous materials (e.g. *a*-Si, *a*-SiO_2_)^25−27^ and amorphous metal–organic frameworks (e.g. *a*-ZIF).^28^ In each of those cases, variation in temperature and/or pressure can certainly drive crystallization—and hence network ordering—but the node arrangements necessarily reorganize in the process.^29,30^ In other aperiodic systems, such as incommensurately modulated crystals, transitions between aperiodic and periodic states are well-known and generally involve switching on and off the relevant modulation.^31,32^ However, in these cases, the parent (ordered) structure has *higher* symmetry than the aperiodic daughter—i.e. the opposite of the behavior in TRUMOF-1. Probably the closest analogy is actually to the emergence of long-range order from spin-ice states in some frustrated magnets^33^ and, by extension, to the reorganization of water molecular orientations across order/disorder transitions in ices themselves.^34,35^

We have long held the belief that TRUMOF-1 is unlikely to be the only Truchet-tile MOF, and our discovery that it forms as a metastable intermediate en route to a “conventional” MOF suggests that *in situ* diffraction studies may offer a useful tool with which to discover other Truchet-tile systems. Perhaps the very low symmetry of msw-TRUMOF-1 hints at a useful search criterion for identifying other TRUMOF candidates: it may prove worthwhile to track *in situ* the crystallization process of other low-symmetry (conventionally) crystalline MOFs,^36,37^ especially when they are assembled from chemically simple components. One way or the other, access to msw-TRUMOF-1 allows us now to understand the effect of topological (dis)order on a variety of physical and chemical properties, including morphology, mechanical response,^38^ dynamics, microporosity, and chemical stability, and we intend to study these various aspects in the near future.

## Supporting Information Available

Details of *in situ* diffraction measurements, determination of phase formation kinetics, and structure solution of msw-TRUMOF-1 (PDF)
