# Supplementary material for: In Situ Observation of Topotactic Linker Reorganization in the Aperiodic Metal–Organic Framework TRUMOF-1
Source: J Am Chem Soc. 2024 Sep 26;146(40):27262–6. doi: 10.1021/jacs.4c09487 (PMC11467960; doi:10.1021/jacs.4c09487)
Supplement: Supplementary file 1 — ja4c09487_si_001.pdf [file ja4c09487_si_001.pdf]

In situ observation of topotactic linker reorganisation in the aperiodic metal–organic framework TRUMOF-1

SUPPLEMENTARY INFORMATION

Guy Greenbaum,[a] Patrick W. Doheny,[b] Robert A. I. Paraoan,[a] Yevheniia Kholina,[c] Stefan Michalik,[d] Simon J. Cassidy,[a] Hamish H.-M. Yeung[b*] and Andrew L. Goodwin[a*]

[a]Department of Chemistry, University of Oxford, Inorganic Chemistry Laboratory, Oxford OX1 3QR, U.K.

[b]School of Chemistry, University of Birmingham, Birmingham B15 2TT, U.K.

[c]Department of Materials, ETH Zürich, 8093 Zürich, Switzerland

[d]Diamond Light Source Ltd., Harwell Science and Innovation Campus, Didcot OX11 0DE, U.K.

*To whom correspondence should be addressed;

E-mail: h.yeung@bham.ac.uk; andrew.goodwin@chem.ox.ac.uk

Contents

1 *In situ* **diffraction measurements** **3**

2 Phase formation kinetics **6**

3 Structure solution of msw-TRUMOF-1 **10**

4 References **17**

1 *In situ* diffraction measurements

Experimental setup

TRUMOF-1 was prepared by a solvothermal reaction, carried out at three separate temperatures. For each temperature measurement, a mixture of zinc nitrate hexahydrate (1.32 mmol, 392 mg) and 1,3-benzenedicarboxylic acid (1.32 mmol, 220 mg) was placed in a 5 ml borosilicate glass culture tube fitted with a PTFE-lined screw cap. Following addition of the solid precursors, a magnetic stirrer bar was added to the vessel, followed by *N*,*N*-dimethylformamide (DMF; 5 ml, 4.72 g). The reaction vessel was sealed, placed in a metal block atop a hotplate stirrer, and then heated to a chosen fixed temperature between 100 and 130 °C (note the boiling point of DMF is 153 °C). Further details regarding this heating cell are available in Ref. S 1 and from the I12 website (`https://www.diamond.ac.uk/Instruments/Imaging-and-Microscopy/I12.html`). Our choice of reaction temperatures was informed by the value used in Ref. S2 (*viz.* 110 °C). In all cases, TRUMOF-1 formation was observed and the reaction was stopped after a fixed period. The metal block was designed with a narrow hole to allow for X-ray beams to penetrate easily through the vessel. On the other side of the heating block there was a cone-shaped hole to allow for diffracted X-rays to leave.

Synchrotron X-ray diffraction measurements were carried out using the I12 beamline at the Diamond Light Source.[S6] Diffraction data were collected in transmission mode using a flat panel detector Pilatus CdTe 2M.[S3] An X-ray energy, sample-to-detector distance, detector geometry in respect to a X-ray beam were determined by measuring a NIST CeO_2 powder standard at multiple sample-to-detector distances.[S4] The X-ray wavelength used was 0.22603 Å (54.853 keV), covering an angular range of $0.4 \leq 2\theta \leq 6.0^\circ$. Once the synthesis process was initiated, the vessel was continuously scanned with the X-ray beam of 0.5 mm × 0.5 mm, using an acquisition time of 10 seconds. 2D diffraction images were azimuthally integrated into intensity curves versus 2θ employing the DAWN software.[S5]

Summary of measured data

Film plot representations of the measured diffraction data are given in Fig. S1. A similar reaction profile was observed in each measurement. Bragg reflections associated with the $F\bar{4}3m$ phase of TRUMOF-1 emerged after an initial incubation period. The TRUMOF-1 phase was then stable for approximately the same length of time as incubation itself. Eventually, a new set of Bragg reflections emerged with an overall intensity profile closely related to that of TRUMOF-1. This later-time-period diffraction pattern was

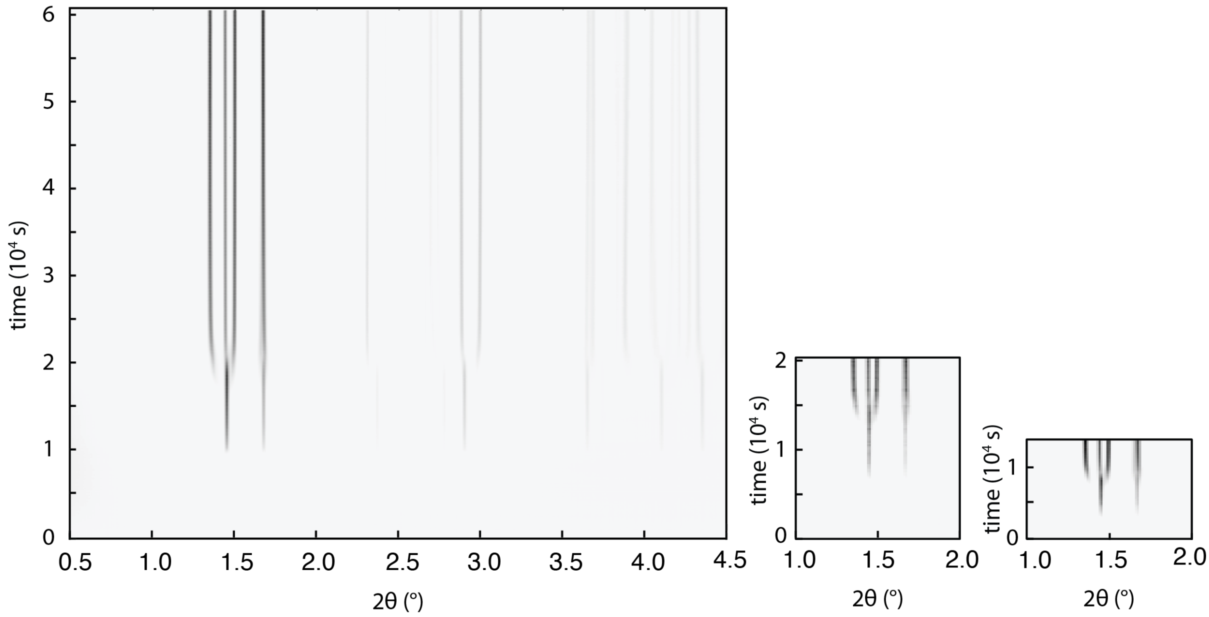

Figure S1: Film plot representations of our *in situ* synchrotron X-ray diffraction measurements, carried out at temperatures of 110 (left), 118 (middle), and 128 °C (right). The diffraction patterns at elevated temperatures are shown over for the most informative 2θ range but measurements were made of the same full 2θ range shown for 110 °C. The film plots show three primary regimes: an initial incubation period, followed by TRUMOF-1 nucleation and growth, and finally transformation to the low-symmetry msw-TRUMOF-1 structure at longer times. This reaction profile occurs at accelerated rates for higher temperatures. All the major peaks in the new phase can be related to those of TRUMOF-1, which suggests that the two structures are linked.

noticeably more complex than that of TRUMOF-1, indicating that it arose from a phase of substantially lower crystal symmetry. This low symmetry phase—later identified as msw-TRUMOF-1—remained stable until the end of each measurement. The incubation and TRUMOF-1 stability time periods decreased systematically as reaction temperature increased.

We show in Fig. S2 a representative constrained Rietveld refinement carried out for the $F\bar{4}3m$ TRUMOF-1 phase using X-ray diffraction data collected *in situ* at 110 °C. In this refinement, which was performed using the TOPAS software,[S7] atom positions and occupancies were fixed to the values determined using single-crystal X-ray diffraction measurements as reported in Ref. S2. The unit-cell, peak-shape and background parameters were refined. All atoms were assigned a single common isotropic displacement parameter, which was also refined. We found evidence of remnant electron density centred around the $4f$ Wyckoff position, which is the centre of the largest pore of the TRUMOF-1 structure. Consequently we included in our refinement an additional dummy atom (we used Xe), with fixed isotropic

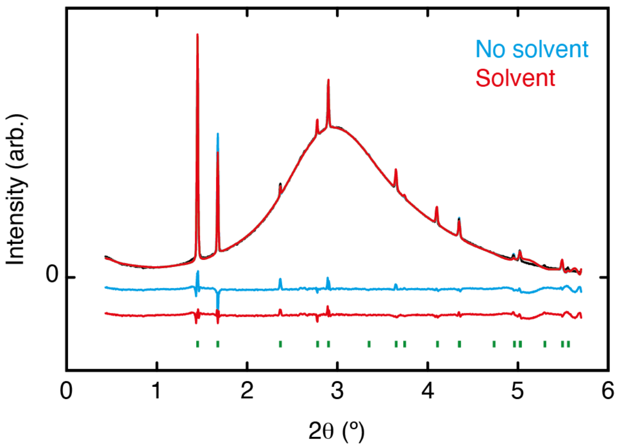

Figure S2: Synchrotron X-ray diffraction pattern of TRUMOF-1 collected *in situ* during crystallisation at 110 °C (black lines), together with constrained Rietveld fits excluding (blue lines) and including (red lines) dummy solvent atoms on the $4f$ Wyckoff positions of $F\bar{4}3m$. The calculated reflection positions are shown as green tick marks, and the difference functions (data − fit) are shown as red and blue lines vertically shifted from the experimental data.

displacement parameter ($B_{\mathrm{eq}} = 2\,\text{Å}^2$) but refineable occupancy. The final lattice parameters and solvent electron counts per unit cell are given in Table S1.

Table S1: Summary of key refined and refinement parameters obtained for the TRUMOF-1 phase observed during *in situ* crystallisation experiments. The parameter e_{solv} denotes the number of electrons associated with occupancy of dummy solvent atom positions.

Temperature (°C)	a (Å)	e_{solv} / u.c.	R_{wp} (%)
110	15.4701(4)	57.0(19)	1.25
118	15.5256(14)	41(6)	1.16
128	15.5087(6)	56(3)	1.35

2 Phase formation kinetics

Sequential Pawley refinements

We monitored the temporal phase evolution throughout our *in situ* measurements using a two-phase constrained Pawley refinement, carried out as follows. Our first step was to identify the very latest diffraction pattern that contained contributions only from TRUMOF-1. We then carried out a Pawley refinement against these data, using $F\bar{4}3m$ as the corresponding space group and fixing the scale factor to unity. The reflection intensities, peak-shape parameters, and unit-cell parameter so obtained were fixed for all subsequent refinements for this TRUMOF-1 phase. In a similar spirit, we used the very last (longest time-scale) diffraction pattern to carry out a similar Pawley refinement for the msw-TRUMOF-1 phase. Since the unit-cell metric and systematic absences of this phase could be well accounted for using a Pm cell (see Section 3 below for further discussion), we used this monoclinic cell for our Pawley refinement. Again, the scale factor was set to unity, and the corresponding cell parameters, peak shape parameters, and reflection intensities were refined in order to obtain the best possible fit to this very last data set. For subsequent refinements, we used a two-phase model containing both the TRUMOF-1 and msw-TRUMOF-1 phases. The phase fractions of each component were allowed to refine, as were the unit cell parameters of the msw-TRUMOF-1 phase, but the reflection intensities for both phases were fixed. This level of constraint was necessary because some peaks overlapped between TRUMOF-1 and msw-TRUMOF-1 phases. Also, because the msw-TRUMOF-1 phase emerged with broader reflections, we included for this phase a crystallite size model, which contributed a single additional parameter that was an estimation to the crystallite size contribution to peak broadening; we included this for the msw-TRUMOF-1 phase but not for TRUMOF-1 itself (since the corresponding peak widths showed no meaningful variation with time). The resulting phase fractions, lattice parameters, and (inverse) crystallite sizes are shown in Fig. S3 for all three temperature points explored experimentally.

The same qualitative behaviour was observed at all three temperature points, albeit that the timescales involved were reduced as temperature is increased. When converting these data to those shown in Fig. 1(b) of the main text, we renormalised the phase fractions by the integrated intensities for TRUMOF-1 and msw-TRUMOF-1 phases determined using the known structure solutions.

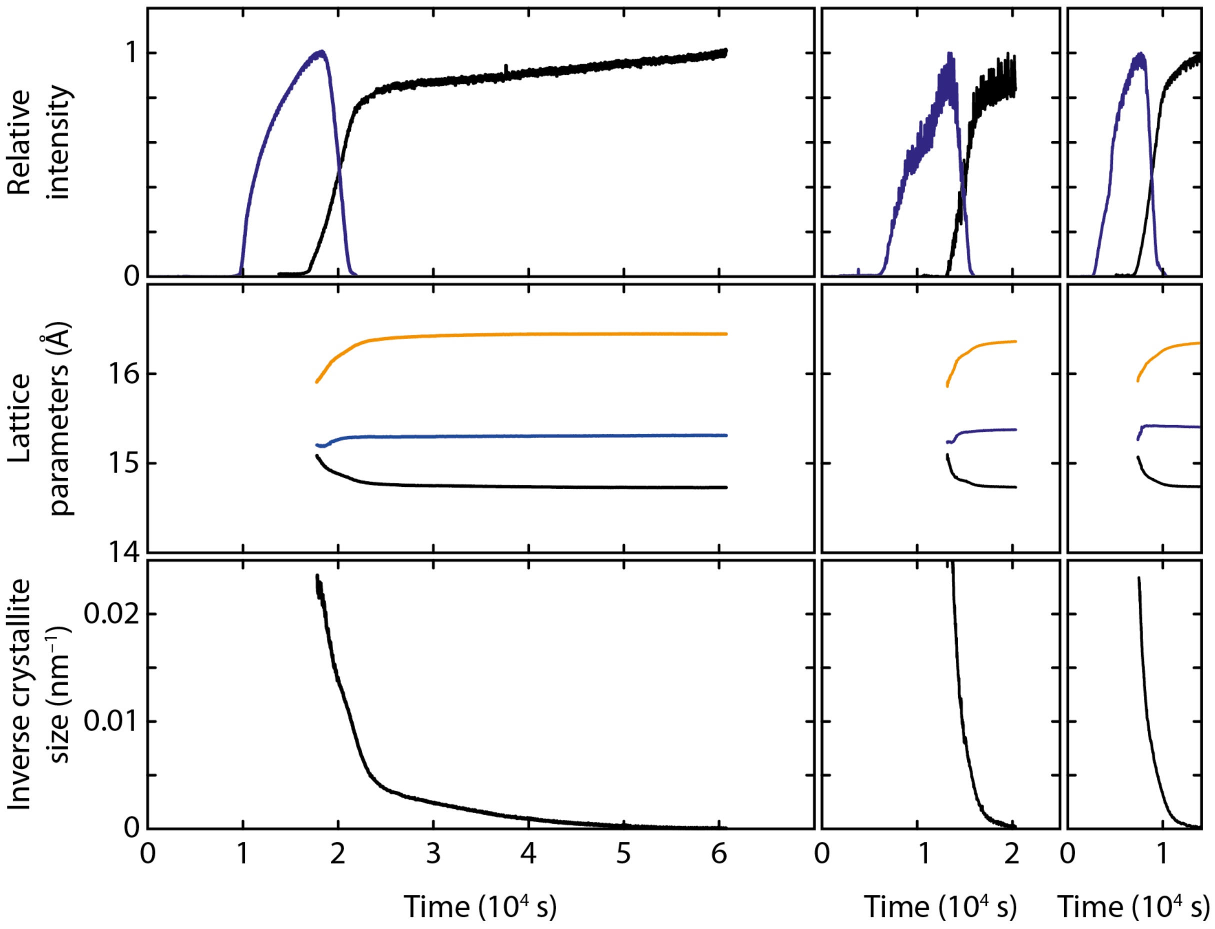

Figure S3: Evolution of key structural parameters during *in situ* crystallisation experiments, extracted from the raw synchrotron X-ray diffraction measurements of Fig. S2 using Pawley refinement. The top panels show the relative fractions of TRUMOF-1 (blue) and msw-TRUMOF-1 (black) phases, scaled to a common maximum value. The middle panels show the reduced lattice parameters of msw-TRUMOF-1: $a_{\rm eff}$ (blue), $b_{\rm eff}$ (grey), and $c_{\rm eff}$ (orange). The bottom panels show the inverse crystallite size of the msw-TRUMOF-1 phase.

Modified Gaultieri fits

Access to time-dependent phase fractions allowed us to explore the nucleation and growth kinetics of both TRUMOF-1 and msw-TRUMOF-1 phases (also as a function of temperature). Because there were three regimes to be considered, we used a rather complex model containing the following ingredients. First, the early time-dependence of the TRUMOF-1 phase fraction α was modelled using the Gualtieri formalism[S8]

$$\alpha(t) = \{1 - \exp[-(k_{\rm g}t)^{n_{\rm g}}]\} \times \frac{1}{1 + \exp[-(t - a_{\rm n})/b_{\rm n}]}, \qquad (1)$$

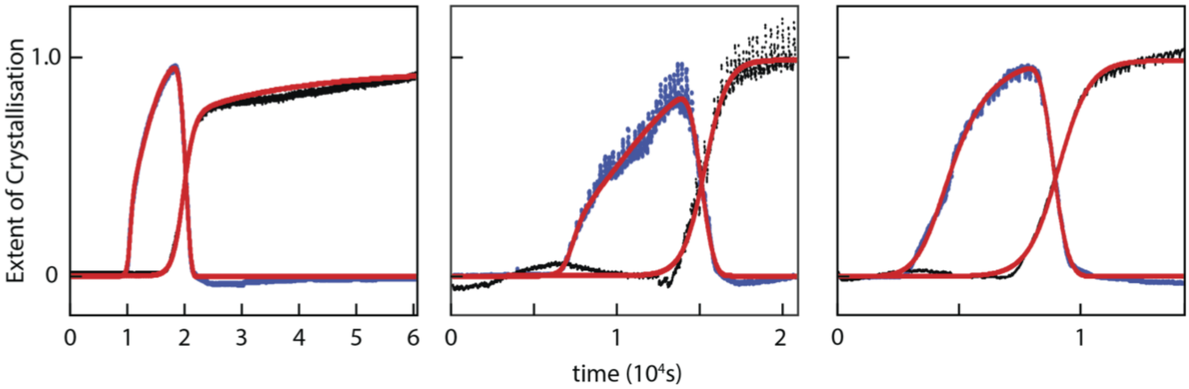

Figure S4: Kinetic fits to the data at three temperatures (110, 118, and 128 °C, from left to right). The blue and grey lines are the extent of crystallisation for TRUMOF-1 and msw-TRUMOF-1 phases respectively, obtained by the two-phase refinement. The red lines are the calculated Gualtieri fits to the data.

where $a_{\rm n}$ and $b_{\rm n}$ are the mean nucleation time and distribution width, respectively, and $k_{\rm g}$ and $n_{\rm g}$ are the growth rate and critical exponents respectively. The eventual disappearance of TRUMOF-1 was incorporated by modifying Eq. (1) with a time-reversed Avrami–Erofe'ev term[S9]

$$\alpha'(t) = \alpha(t) \times \left(1 - \exp\{-[k_{\rm d}(t - t_{\rm d})]^{n_{\rm d}}\}\right), \tag{2}$$

where $k_{\rm d}$ and $n_{\rm d}$ are disappearance rate and critical exponents, respectively. The emergence of the msw-TRUMOF-1 phase was modelled using its own Gaultieri expression.

Consequently, for each temperature point, the ensemble of data was fitted using a total of nine parameters. Those parameters associated with growth kinetics could not be determined with any great confidence because none of the growth processes extended to completion (the Gualtieri parameters being especially sensitive to this). In the case of msw-TRUMOF-1 this situation was so poorly constrained that we fixed the relevant parameters to a common constant value; the choice of this value made very little difference to the eventual fits. We show in Fig. S4 the resulting fits obtained, and list in Table S2 the corresponding model parameters obtained by these fits to our variable-temperature data. The corresponding activation energies obtained were 61(3), 30(20), and 57(4) $\mathrm{kJ\,mol^{-1}}$ for TRUMOF-1 nucleation, TRUMOF-1 growth, and msw-TRUMOF-1 nucleation, respectively.

Table S2: Kinetic parameters obtained for TRUMOF-1/msw-TRUMOF-1 nucleation and growth. Those parameter values given in italics were fixed because of a correspondingly low sensitivity in the data.

Temperature (°C)		110	118	128
TRUMOF-1	k_{g} (10^{-5} s^{-1})	7.75(2)	9.100(13)	22.2(5)
	n_{g}	3.54(5)	2.81(3)	2.3(2)
	a_{n} (s)	10 252(9)	6980(20)	3840(80)
	b_{n} (s)	291(7)	326(18)	619(13)
	k_{d} (10^{-4} s^{-1})	4.67(8)	5.0(2)	4.9(2)
	n_{d}	2.66(7)	3.6(2)	4.9(3)
	t_{d} (10^{3} s)	21.90(4)	16.50(10)	10.6(10)
msw-TRUMOF-1	k_{g} (10^{-3} s^{-1})	*1*	*1*	*1*
	n_{g}	*3*	*3*	*3*
	a_{n} (s)	20 262(13)	14 920(10)	8939(5)
	b_{n} (s)	1211(11)	656(9)	630(4)

3 Structure solution of msw-TRUMOF-1

Indexing of PXRD trace

Our starting point for determining the structure of the long-timescale phase was to recognise the similarity in diffraction intensity profile to that of TRUMOF-1 itself. This similarity implied the likelihood of a group–subgroup relationship between the two structure types. The lowest-angle reflection of the $F\bar{4}3m$ TRUMOF-1 phase is the (111) reflection ($2\theta \simeq 1.4^\circ$ in Fig. S2). On phase transformation, this peak splits into (at least) three peaks. Given the multiplicity of the (111) reflection (eight), this splitting is consistent with only with descent to either monoclinic or triclinic symmetry. The highest-symmetry monoclinic subgroup of $F\bar{4}3m$ that splits this peak appropriately has Cm space-group symmetry and a cell volume half that of the $F\bar{4}3m$ cell.[S10] A Pawley refinement carried out using this Cm cell gave a good fit to data, but was unable to account for any intensity at a handful of small superlattice reflection positions (forbidden in Cm) [Fig. S5]. We found that all these remaining reflections could be accounted for by removal of the lattice centering operation, corresponding to a symmetry reduction to the monoclinic space-group Pm. The relationship between $F\bar{4}3m$, Cm, and Pm cells is shown in Fig. S6.

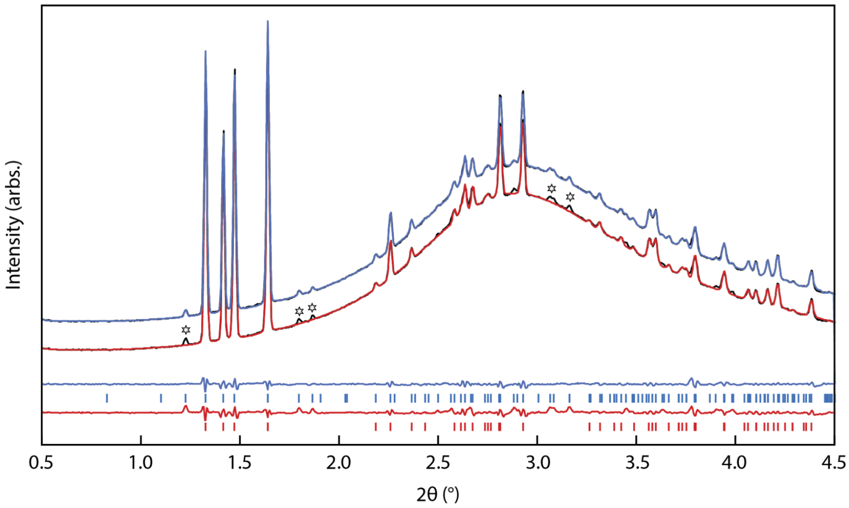

Figure S5: Two different Pawley fits to the same diffraction pattern of the long-timescale phase. The first fit (red) was refined in Cm but missed the starred peaks. The second refinement (blue) was carried out in the Pm space group, and gave rise to a a near-perfect fit with all peaks being accounted for.

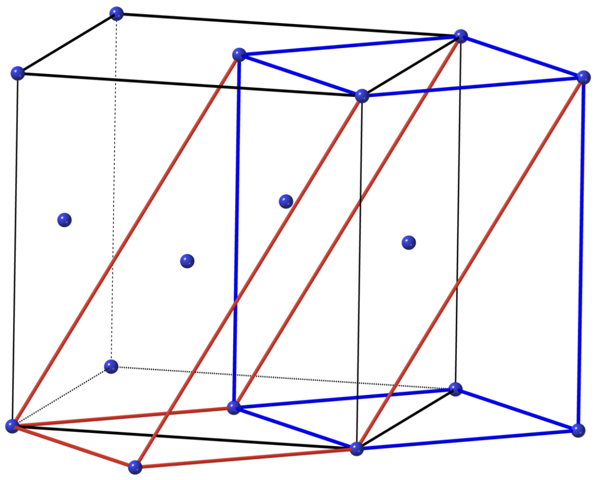

Figure S6: Figure illustrating the location of the Cm (red) and Pm (blue) cells explored when indexing the X-ray diffraction pattern of the long time-scale phase, shown relative to the original $F\bar{4}3m$ cell of TRUMOF-1 (black). The original lattice points (at which OZn_4 clusters are located) are shown as blue spheres.

Initial Pm solution

Having shown that the long timescale X-ray diffraction pattern could be indexed according to a Pm cell, we proceed to attempt a Rietveld refinement against our experimental data. Our starting point was to use the original $F\bar{4}3m$ TRUMOF-1 structure,[S2] transformed into this new low-symmetry cell, as a preliminary structural model. The corresponding unit-cell contained two symmetry-distinct OZn_4 clusters and eight partially-occupied linker sites (six of which were symmetry-inequivalent). Taking into account the symmetry operations present at each linker site, we developed a Rietveld model that allowed simultaneous refinement of the cluster positions/orientations, the linker positions, and the population of different linker geometries. We added degrees of freedom to our model in a sequential manner, but always made sure that the total stoichiometry was appropriate. We also included dummy atoms to account for included solvent. Illustrations of the progressive improvement in quality of fit are shown in Fig. S7.

While the eventual structural model provided an excellent fit to data, the number of parameters was relatively large, and there were a handful of unphysical aspects in the corresponding structural model. For example, the linker occupancies were such that the total coordination of the two OZn_4 was substantially inequivalent. A second problem was that the translations of some linkers was sufficiently large that unphysical bond lengths and bond angles had developed. Another problem was that the linker occupancies at some sites exceeded unity. We did of course explore introducing various additional constraints

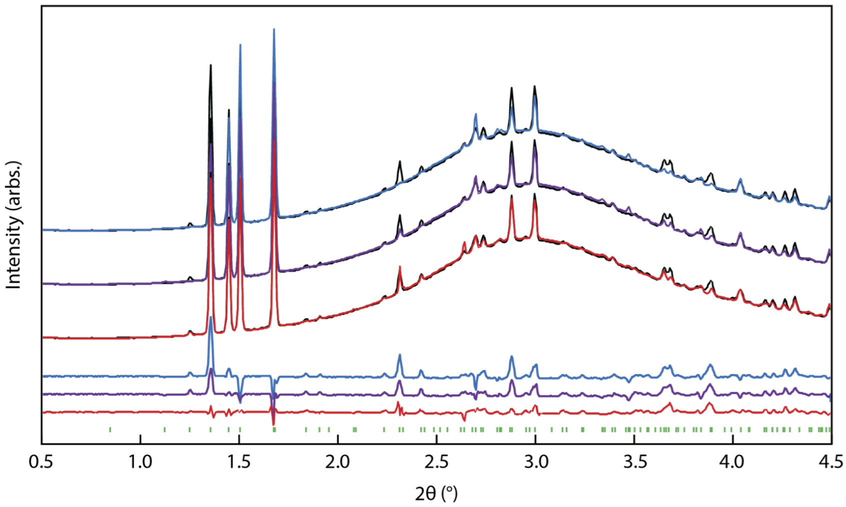

Figure S7: Rietveld fits to X-ray diffraction data collected for the long time-scale phase using models with increasingly many degrees of freedom. The first fit (shown in blue) was based on the atomic coordinates of the original TRUMOF-1 structure but allowed refinement of lattice parameters and isotropic displacement parameters (all atoms have been given the same displacement parameter). The second fit (shown purple) used a more complex model in which the occupancies of 1,3-bdc linkers were allowed to refine, in a way that ensured both Pm symmetry and overall stoichiometry were maintained. The third fit (shown in red) was obtained by adding to this more complex model the inclusion of rigid-body translations of the nodes and linkers.

to try to remedy these problems, but found that there was a large number of different competing models that could give rise to fits of essentially similar quality (so long as sufficiently many parameters were involved).

As stated in the main text, a common feature of these various models was always that a subset of linker occupancies refined to zero. These linkers involved carboxylates pointing along a single common direction (perpendicular to the unique $\mathbf{b}$ axis of the Pm cell). We used the FINDSYM routine in ISODISTORT[S10] to show that omission of a single common carboxylate position from the original $F\bar{4}3m$ cell resulted in a structural model with Cm symmetry—and the Cm unit-cell metric identified above. This symmetry analysis does not of course take into account the correlations between linker occupancies expected from the matching rules identified in Ref. S2.

Consequently we modified the Monte Carlo algorithm of Ref. S 2, originally used to generate TRUMOF-1 configurations, such that it was forbidden from generating configurations with carboxylates occupying a single one of the 12 possible directions. While a variety of disordered configurations could be found to accommodate this rule, only a single solution was found consistent with the $1 \times 1 \times 1$ metric

of the original $F\bar{4}3m$ cell. (This is in contrast to the unconstrained approximant generation, for which three symmetry-distinct $1 \times 1 \times 1$ solutions emerge). This new configuration, which represents the simplest solution satisfying at once both the underlying connectivity rules of TRUMOF-1 and also the new requirement to avoid occupying one common carboxylate position, had $P1$ symmetry with a unit cell of the same metric as the Pm cell used in Pawley refinements above. The reflection conditions for $P1$ and Pm are identical, of course. We note that this solution was one of the $1 \times 1 \times 1$ approximants identified in Ref. S2, and the DFT-optimised structure so determined showed such strong pseudosymmetry that the cell axes were effectively monoclinic.

Final $P1$ Rietveld refinement

Our approach to determine a sensible structural model of the long time-scale phase was then to take the cell metric determined using Pawley refinements, and the atomic positions obtained in DFT optimisation of the relevant $1 \times 1 \times 1$ approximant to generate an initial structural model. The quality of fit-to-data for this model, obtained before any subsequent refinement, is shown in Fig. S8. Given that this model has relatively few free parameters, and is derived from a simple set of chemical rules, we already considered this fit to be good and more physically meaningful than those obtained in the Pm refinements described above.

We proceeded to improve the quality of fit-to-data by allowing the positions and orientations of OZn_4 tetrahedra to refine, and also the positions of each 1,3-bdc linker to refine (again as a rigid body). We ensured sensible chemical connectivity by including bond-length constraints on the Zn–O linkages, and bond-angle constraints for the coordination environments around each Zn centre. Fourier difference maps indicated the presence of disordered solvent within the pore structure. We placed dummy atoms near the midpoints of the two primary maxima in this remnant electron density and refined the corresponding electron count (but not positions). Our final structural model then included 12 distance constraints and 24 angle constraints, treated all OZn_4 and 1,3-bdc units as rigid bodies, and had relatively few internal degrees of freedom. So, while the quality of fit shown in Fig. 3(a) of the main paper is poorer than that of *e.g.* the best in Fig. S7, we consider the former to be more meaningful because it is vastly more constrained and arises from sensible chemical rules. Moreover, the final structural model gives chemically sensible bond geometries and occupancies.

A crystallographic information file (`.cif`) is included as supporting information, but we summarise in Table S3 the unit-cell parameters obtained from our refinement, where we also compare these against

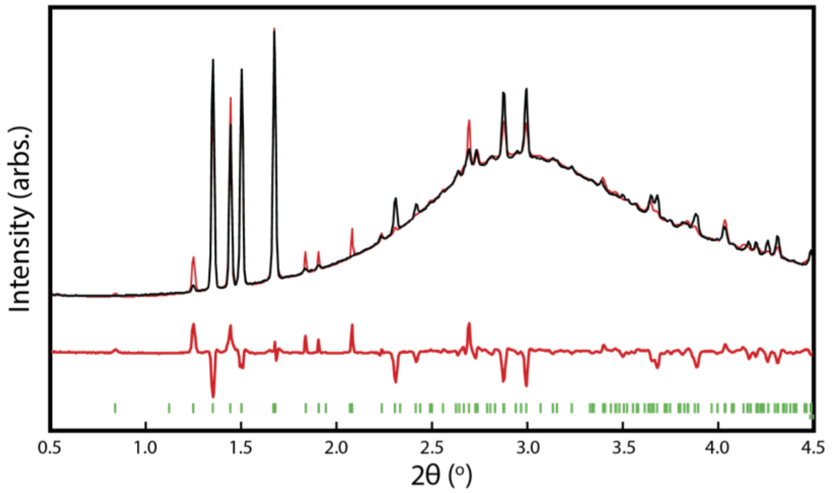

Figure S8: Fit-to-data for the long-timescale X-ray diffraction pattern obtained using the approximant model of Ref. S2, with unit-cell metric taken from our Pm Pawley refinements. While there remain large errors, the key point is that no internal structural reorganisation has yet taken place, so this model carries with it the chemically-sensible bonding coordinations determined using DFT optimisation. Subsequent refinement led to the (improved) fit-to-data shown in Fig. 3(a) of the main text.

Table S3: Crystallographic details for msw-TRUMOF-1 as determined from our constrained Rietveld refinement, and compared against the DFT-optimised approximant description given in Ref. S2. Note that the approximant cell has been transformed to its highest symmetry setting.

	msw-TRUMOF-1	Approximant
Crystal system	Triclinic	Triclinic
Space group	$P1$	$P1$
a (Å)	11.6314(18)	10.5179
b (Å)	10.4162(15)	10.6805
c (Å)	15.532(4)	15.4518
α (°)	90	89.892
β (°)	86.048(7)	87.466
γ (°)	90	89.879
V (Å^3)	1877.305	1734.093

the DFT-optimised approximant reported in Ref. S2. An overlay of the two structures is shown in Fig. S9.

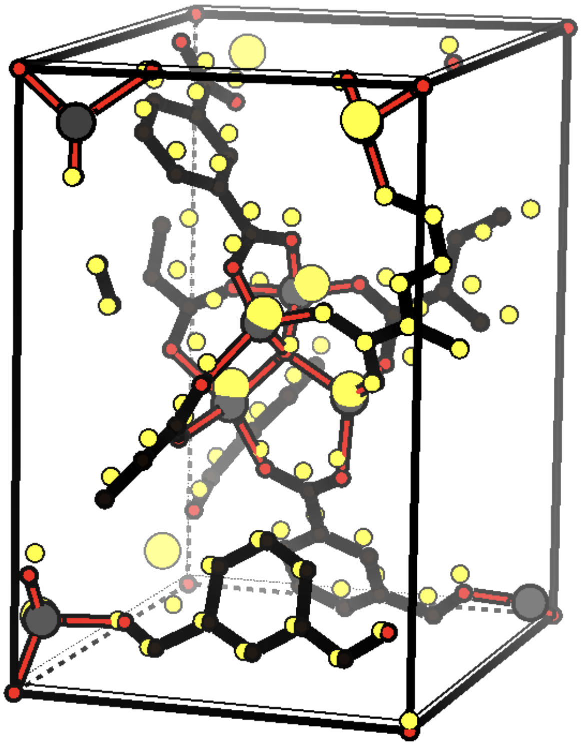

Figure S9: An overlay of the atom coordinates within our final Rietveld model (grey, red, and black atoms) and the DFT-relaxed approximant structure of Ref. S2 (yellow atoms).

Summary of msw topology

We used the toposcryst.com web implementation of the ToposPro package[S11] to identify the network topology of msw-TRUMOF-1. The **msw** net is a uninodal six-connected net whose most symmetry embedding in three-dimensional space has tetragonal $P4_2/nnm$ space-group symmetry.[S12] It is related to a distorted version of the doubly-interpenetrated diamondoid net, where the fourfold connectivity of each node is extended to six by connecting nodes related by a a single common lattice vector. A representation of the high-symmetry net is given in Fig. S10.

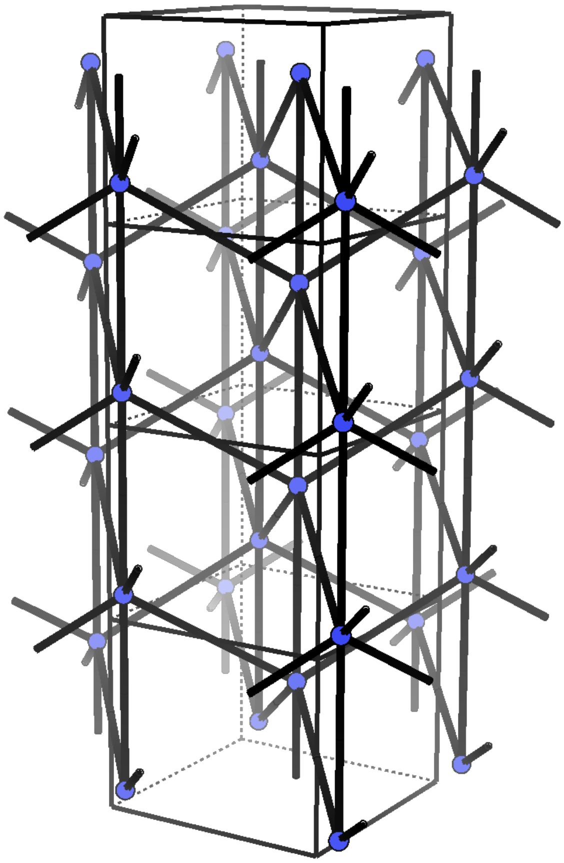

Figure S10: Representation of the **msw** net, with nodes shown as blue spheres and edges as solid black rods. The connectivity of msw-TRUMOF-1 is given by this net.

4 References

(S1) Orr, K. W. P.; Collins, S. M.; Reynolds, E. M.; Nightingale, F.; Boström, H. L. B.; Cassidy, S. J.; Dawson, D. M.; Ashbrook, S. E.; Magdysyuk, O. V.; Midgley, P. A.; Goodwin, A. L.; Yeung, H. H.-M. Single-step synthesis and interface tuning of core–shell metal–organic framework nanoparticles. *Chem. Sci.* **12**, 4494-4502 (2021).

(S2) Meekel, E. G.; Schmidt, E. M.; Cameron, L. J.; Dharma, A. D.; Windsor, H. J.; Duyker, S. G.; Minelli, A.; Pope, T.; Orazio Lepore, G.; Slater, B.; Kepert, C. J.; Goodwin, A. L. Truchet-tile structure of a topologically aperiodic metal–organic framework. *Science* **379**, 357-361 (2023).

(S3) Šišak Jung, D.; Donath, T.; Magdysyuk, O.; Bendarcik, J. High-energy X-ray applications: current status and new opportunities. *Powder Diffraction* **32**, S22-S27 (2018).

(S4) Hart, M. L.; Drakopoulos, M.; Reinhard, C.; Connolley, T. Complete elliptical ring geometry provides energy and instrument calibration for synchrotron-based two-dimensional X-ray diffraction. *J. Appl. Cryst.* **46**, 1249-1260 (2013).

(S5) Filik, J.; Ashton, A. W.; Chang, P. C. Y.; Chater, P. A.; Day, S. J.; Drakopoulos, M.; Gerring, M. W.; Hart, M. L.; Magdysyuk, O. V.; Michalik, S.; Smith, A.; Tang, C. C.; Terrill, N. J.; Wharmby, M. T.; Wilhelm, H. Processing two-dimensional X-ray diffraction and small-angle scattering data in DAWN 2. *J. Appl. Cryst.* **50**, 959-966 (2017).

(S6) Drakopoulos, M.; Connolley, T.; Reinhard, C.; Atwood, R.; Magdysyuk, O.; Vo, N.; Hart, M.; Connor, L.; Humphreys, B.; Howell, G.; Davies, S.; Hill, T.; Wilkin, G.; Pedersen, U.; Foster, A.; De Maio, N.; Basham, M.; Yuan, F.; Wanelik, K. I12: the Joint Engineering, Environment and Processing (JEEP) beamline at Diamond Light Source. *J. Synchrotron Radiat.* **22**, 828-838 (2015).

(S7) Coelho, A. A; *TOPAS-Academic, version 6 (computer software)*, Coelho Software, Brisbane.

(S8) Gualtieri, A. F. Synthesis of Sodium Zeolites from a Natural Halloysite. *Phys. Chem. Miner.* **28**, 719-728 (2001).

(S9) Yeung, H. H.-M.; Wu, Y.; Henke, S.; Cheetham, A. K.; O'Hare, D.; Walton, R. I. In Situ Observation of Successive Crystallizations and Metastable Intermediates in the Formation of Metal–Organic Frameworks. *Angew. Chem. Int. Ed.* **55**, 2012-2016 (2016).

(S10) Stokes, H. T.; Hatch, D. M.; Campbell, B. J. *ISODISTORT*, ISOTROPY Software Suite, iso.byu.edu.

(S11) Blatov, V. A.; Shevchenko, A. P.; Proserpio, D. M. Applied topological analysis of crystal struc-

tures with the program package ToposPro. *Cryst. Growth Des.* **14**, 3576-3586 (2014).

(S12) Delgado-Friedrichs, O.; Foster, M. D.; O'Keeffe, M.; Proserpio, D. M.; Treacy, M. M. J.; Yaghi, O. M. What do we know about three-periodic nets? *J. Solid State Chem.* **178**, 2533-2554 (2005).
